# Papillary thyroid carcinoma: ≤ 10 mm does not always mean pN0. A multicentric real-world study

**DOI:** 10.1007/s13304-024-01779-6

**Published:** 2024-03-06

**Authors:** Stefano Amendola, Tommaso Piticchio, Lorenzo Scappaticcio, Sium Wolde Sellasie, Salvatore Volpe, Rosario Le Moli, Luigi Coppola, Leo Guidobaldi, Francesco Pedicini, Carla Carbone, Paola Caruso, Elena Gamarra, Giovanni Docimo, Francesco Frasca, Luigi Uccioli, Pierpaolo Trimboli

**Affiliations:** 1 Division of Endocrinology and Diabetes, CTO Andrea Alesini Hospital, Department of Biomedicine and Prevention, University of Rome Tor Vergata, 00133 Rome, Italy; 2 Endocrinology Section, Department of Clinical and Experimental Medicine, Garibaldi Nesima Hospital, University of Catania, Catania, Italy; 3 Servizio Di Endocrinologia E Diabetologia, Ospedale Regionale Di Lugano, Ente Ospedaliero Cantonale (EOC), Lugano, Switzerland; 4 Division of Endocrinology and Metabolic Diseases, AOU University of Campania “Luigi Vanvitelli”, Naples, Italy; 5 UOC of Pathologic Anatomy and Cytodiagnostic, Sandro Pertini Hospital, ASL RM2, 00157 Rome, RM Italy; 6 Thyroid Endocrine Surgery, Sant’Eugenio Hospital, 00144 Rome, Italy; 7 Division of Thyroid Surgery, University of Campania “L. Vanvitelli”, Naples, Italy; 8 Faculty of Biomedical Sciences, Università Della Svizzera Italiana (USI), Lugano, Switzerland

**Keywords:** Thyroid, Papillary, Thyroid microcarcinoma, Lymph node metastasis

## Abstract

The incidence of papillary thyroid carcinoma (PTC) is increasing and PTC ≤ 10 mm (PTMC) accounts for most new diagnoses. PTMCs are not always low risk, as detection of lymph nodes metastasis (LNM) may occur. The purpose of the study was to analyze the clinical pattern, frequency, and independent risk factors of patients with PTMC and LNM. From January 2022 to June 2023, PTCs managed at CTO Hospital, Rome; Policlinico Vanvitelli, Naples; and Garibaldi Nesima Hospital, Catania were included. PTC management followed the same diagnostic–therapeutic procedures according to the ATA guidelines. Variables such as age, sex, maximum diameter, histologic evidence of LNM (HELNM +), Hashimoto’s thyroiditis (HT), multifocality, capsule invasion, and histological subtype were considered. PTCs were divided according to HELNM and size. Two hundred ninety-eight PTCs were included. PTMCs were 136 (45.6%) and LNM occurred in 27.2% of them. In the HELNM + group, analysis of PTMC vs ‘MacroPTC’ (PTC > 10 mm) did not show any statistical difference. Multivariate regression revealed that young age (OR 0.93; CI 95% 0.90–0.96; *p* < 0.01) and male sex (male OR 3.44; CI 95% 1.16–10.20; *p* = 0.03) were the only independent risk factors for HELNM + in PTMC. The risk of LNM in PTMC is not negligible; therefore, a careful evaluation by an expert thyroidologist is mandatory for patients with small thyroid nodule, especially in younger and male patients before excluding surgery. In the future, new tools are needed to detect early PTMC with LNM before surgery.

## Introduction

Thyroid carcinoma (TC) is the most common endocrinological malignancy and is one of the ten most common worldwide tumors. Among TCs, the most common histological subtype is papillary thyroid carcinoma (PTC), which accounts for up to 96% [1]. In recent years, the incidence of TC has increased, driven by detection of small PTCs [2]. Many authors have attributed these figures to the widespread use of neck ultrasound (NEUS) in clinical practice, also according to non-thyroid indication. However, new epidemiological, biological, and clinical data from the SEER program appear to suggest a true biological increase that contributes to the overall increase of TC incidence [3]. This phenomenon can be attributed to several factors such as radiation exposure, iodine intake, environmental pollution, and modern dietary habits [4]. Anyway, thyroid nodule, and therefore TC, is a health problem in terms of psychological burden, decreased quality of life, personal and social costs of treatment, and surgical complications.

Taking into account the above issues, to lower the burden on institutions, physicians, and patients, scientific societies do not recommend performing biopsies in very small thyroid nodules (i.e., maximum diameter less than 10 mm), even if they show suspicious characteristics of the US features [5, 6]. This approach could avoid overdiagnosis and overtreatment of low-risk indolent papillary thyroid microcarcinoma (PTMC), but at the same time, it could also overlook the diagnosis of the aggressive ones in their early stages [7]. In fact, to date, there is no agreement on the clinical characteristics that can reliably discriminate patients with small but aggressive TC from the larger number of indolent PTMC [8–13]. This controversial topic is highlighted in the fifth edition of WHO classification [14]. So, it is no longer recommended to refer to PTMC without histological subtype identification.

PTC can metastasize to neck lymph nodes (LNM) which occur first in central compartment and then in the laterocervical compartment homolateral to the primary thyroid lesion. However, it has also been described to skip metastasis cases in PTC [8]. PTC has generally a good prognosis; however, structural recurrence can occur during post-operative follow-up and LNM was the main risk factor for recurrence in patients with PTC [15]. Several previous studies investigated risk factors for LNM, but did not achieve the same results [8, 16, 17]. Former evidence identified male sex, young age, multifocality [18], and large tumor size as the main risk factors for LNM [16]. Despite this, about age and tumor size, there is no agreement regarding specific cut-offs [19, 20]. Furthermore, in few studies, histological subtypes were considered [21], although their importance was remarked in the last edition of the WHO classification.

Considering these limitations in the current literature and considering the huge number of small thyroid nodules detected in daily clinical practice, the present study was aimed: 1) to analyze the clinical pattern of PTMCs with LNM; and 2) to investigate independent risk factors for LNM in PTMC. To this purpose, data from three high-volume Italian hospitals specialized in thyroid disease institutions were retrospectively analyzed.

## Methods

### Institutional setting and management of thyroid nodules’ patients

Three institutions (CTO Hospital, Rome; Policlinico Vanvitelli, Naples; and Garibaldi Nesima Hospital, Catania) participated in this study. These centers are high-volume institutes for thyroid diseases and apply the same standard diagnostic–therapeutic procedures for patients with thyroid nodules according to the ATA guidelines [15]. All patients who refer to these institutions for thyroid disease undergo a general thyroid hormonal assessment (i.e., TSH, plus FT3 and FT4 when indicated, and Calcitonin) and NEUS performed by endocrinologists with at least 10 years of experience. Thyroid nodules with suspicious of malignancy at NEUS undergo fine needle aspiration cytology (FNAC). Cytological smears are classified according to the Italian guidelines SIAPEC-AIT 2014 [22]. Neck dissection of the central cervical compartment was performed in patients with clinically uninvolved central neck lymph nodes (cN0) who have advanced primary tumors (T3 or T4) or clinically involved central (cN1a) and/or lateral neck nodes (cN1b), by NEUS evaluation according to the ETA cervical US guidelines [23]. When LNM suspicious of the lateral cervical compartment was recognized, FNAC is performed for both cytology and thyroglobulin measurement in needle washout (FNA-Tg). Surgery (total thyroidectomy vs. lobo-isthmectomy) was scheduled close to diagnosis, and the extension of surgery is always according to NEUS, FNAC / FNA-Tg, and individual features.

### Case selection

The institutional database of participating institutions was retrospectively reviewed to select patients who underwent thyroid surgery from January 2022 to June 2023. The inclusion criteria were: (1) age > 18 years; (2) FNAC report of indeterminate, suspicious for or consistent with malignancy [22]; and 3) histological diagnosis of PTC. Exclusion criteria were: (1) thyroid nodule management or surgery in another clinical setting; (2) incidental histological diagnosis of PTC in a patient who underwent surgery for benign thyroid disease; and (3) histological finding of ≤ 5 lymph-node micrometastasis, defined by maximum diameter < 2 mm. All patients signed the informed consent and privacy forms.

### Measure and reference standard

The recruited population was divided into two groups according to histological evidence (HELNM +) or not (HELNM-) of LNM. The HELNM + group was defined by at least one metastatic lymph node in the lateral or central compartments of the neck on histological examination (i.e., pN1). Incidental pathological finding of LNM in peri-thyroid lymph nodes was included in HELNM + group [24]. The HELNM- group included patients who underwent lymph node dissection with pN0 and cases without neck dissection (i.e., pNx). The size of the PTC was classified as follows: ≤ 10 mm defined as ‘PTMC’ and > 10 mm defined as ‘MacroPTC’. Multifocal malignant tumour was defined when multiple unilateral or bilateral foci were discovered. Hashimoto thyroiditis (HT) was assessed on histological examination. Capsule invasion was defined according to ATA guidelines [15]. According to histological subtype, PTCs were divided into aggressive, including tall cell, hobnail, columnar cell, solid/trabecular, and diffuse sclerosing subtype, and not aggressive including classic and follicular. [25].

### Statistical analysis

Age and maximum diameter of the primary lesion were expressed as median and interquartile ranges (IQR) and compared by the Mann–Whitney *U* test. Sex, multifocality, capsule invasion, HT, and histological subtype were expressed as frequencies and compared using the *x*^2^-test. The clinical characteristics related to LNM in PTMC were explored by univariate analyses with logistic models, and then by multivariate logistic models. A *p* value of < 0.05 was considered to define statistical significance. All statistical analyses were performed with Jamovi software version 2.3 retrieved from https://www.jamovi.org.

## Results

A total of 298 patients with PTC were included (that is, 118 at the CTO Hospital, 80 at the Policlinico Vanvitelli and 100 at Garibaldi Nesima Hospital). The mean age of the population was 49.5 years and 232 were women. PTMCs were 136 (45.6%). One hundred and one patients had also histological diagnosis of HT. The median maximum diameter of the PTC was 11 mm. One hundred and ninety-two patients underwent cervical lymphadenectomy (64.5%) of which 59.3% N1 (N1a 39% and N1b 20.3%) and 40.7% N0. Among PTMCs, 58.1% underwent cervical lymphadenectomy and HELNM + occurred in 27.2% of them. Twenty-eight PTCs had aggressive subtype of which 46.4% were PTMC.

Two groups according to the size of the primary lesion (i.e., PTMC versus MacroPTC) were compared with significant differences in age (*p* = 0.02) and prevalence of HELNM + (*p* < 0.01). Descriptive and comparative analyses are reported in Table 1.

**Table 1:** Descriptive and comparative analysis (‘MacroPTC vs PTMC) of PTCs enrolled

	Total	‘MacroPTC’	PTMC	*p*
N. cases	298	162 (54.3%)	136 (45.6%)	
Age (median ± IQR)	49.5 (± 22)	47(± 20)	51(± 22)	0.02
Sex				
Male	66 (22.1%)	43 (26.5%)	23 (16.9%)	0.06
Female	232 (77.9%)	119 (73.5%)	113 (83.1%)	
Maximum diameter (median ± IQR)	11 (± 9)	16(± 10)	7 (± 3)	< 0.01
Multifocality				
Yes	136 (45.6%)	73 (45.1%)	63 (46.3%)	0.83
No	162 (54.4%)	89 (54.9%)	73 (53.7%)	
Capsule invasion				
Yes	161 (54.1%)	92 (56.8%)	69 (50.8%)	0.30
No	137 (45.9%)	70 (43.2%)	67 (49.2%)	
Hashimoto’s thyroiditis				
Yes	101 (33.9%)	49 (30.2%)	52 (38.2%)	0.15
No	197 (66.1%)	113 (69.8%)	84 (61.8%)	
Histological evidence of lymph node metastasis				
Yes (HELNM +)	114 (38.5%)	77 (47.5%)	37(27.2%)	< 0.01
No (HELNM–)	182 (61.5%)	85 (52.5%)	99 (72.8%)	
Histological subtype				
Aggressive	28 (9.4%)	15 (9.3%)	13 (9.6%)	0.93
Not aggressive	270 (90.6%)	147 (90.7%)	123 (90.4%)	

When just HELNM + cases were analyzed, no differences were shown between PTMC and MacroPTC in terms of age (*p* = 0.769), sex (*p* = 0.466), multifocality (*p* = 0.158), capsule invasion (*p* = 0.954), HT (p = 0.790) and histological subtype (*p* = 0.347) (Table 2).

**Table 2:** Comparison between PTMC and MacroPTC among HELNM + cases

	PTMC (27.2% of PTMC)	MacroPTC (47.5% of MacroPTCs)	*p*
Age			
Median	41 (± 20)	38 (± 20)	0.842
Sex			
Male	11	26	0.466
Female	18	59	
Multifocality			
Yes	14	40	0.158
No	23	37	
Capsule invasion			
Yes	23	47	0.954
No	14	30	
Hashimoto’s thyroiditis			
Yes	8	15	0.790
No	29	62	
Histological subtype			
Aggressive	3	11	0.347
Not aggressive	34	66	

PTMC ≤ 10 mm; MacroPTC > 10 mm

Focusing on PTMCs, there were significant differences in terms of HT (*p* = 0.015), sex (*p* = 0.015), maximum diameter (*p* < 0.01), and age (*p* < 0.01) between HELNM + and HELNM– (Table 3).

**Table 3:** Comparison between PTMCs assessed as HELNM + or HELNM-

	HELNM- (99/136)	HELNM + (37/136)	*p*
Age			
Median	56 (± 18)	41 (± 20)	0.001
Maximum diameter (mm)	7 (± 4)	8(± 2)	0.014
Sex			
Male	12	11	0.015
Female	87	26	
Multifocality			
Yes	49	14	0.225
No	50	23	
Capsule invasion			
Yes	14	23	0.101
No	50	43	
Hashimoto’s thyroiditis			
Yes	44	8	0.015
No	55	29	
Histological subtype			
Aggressive	10	3	0.725
Not aggressive	89	34	

N –: absence of LNM, N + : presence of LNM

Univariate logistic regression analyses showed statistical significance for sex (male OR 3.07; CI 95%: 1.21–7.76; *p* = 0.02), age (OR 0.93; CI 95%: 0.90–0.96; *p* < 0.01), maximum diameter (OR 1.25; CI 95%: 1.02–1.52; *p* = 0.03) and HT (yes OR 0.35; CI 95%: 0.14–0.83; *p* = 0.02) for PTMCs with HELNM + (Table 4).

**Table 4:** Univariate analysis of PTMC with/without histological evidence of lymph node metastasis

	Univariate analysis
Age	OR 0.93 (CI 95%: 0.90–0.96) *p* < 0.01
Maximum diameter (mm)	OR 1.25 (CI 95%: 1.02–1.52) *p* = 0.03
Sex	
Male	OR 3.07 (CI 95%: 1.21–7.76) *p* = 0.02
Hashimoto’s thyroiditis	
Yes	OR 0.35 (CI 95%: 0.14–0.83) *p* = 0.02
Multifocality	
Yes	OR 0.62 (CI 95%: 0.29–1.35) *p* = 0.23
Capsule invasion	
Yes	OR 1.91 (CI 95%: 0.88–4.65) *p* = 0.10
Histological subtype	
Aggressive	OR 0.78 (CI 95%: 0.20–3.03) *p* = 0.73

Finally, considering the results of the univariate analyses, a multivariate logistic regression (Table 5) was performed. The best fit model (R^2^_McF_ = 0.192; AIC = 135; *p* < 0.01) included only age (OR 0.93; CI 95%: 0.90–0.96; *p* < 0.01) and sex (male OR 3.44; CI 95%: 1.16—10.20; *p* = 0.03).

**Table 5:** Multivariate analysis of PTMC with/without histological evidence of lymph node metastasis

	Multivariate analysis
Age	OR 0.93 (CI 95%: 0.90–0.96) *p* < 0.001
Sex	
Male	OR 3.44 (CI 95%: 1.16—10.20) *p* = 0.03

## Discussion

The incidence of PTC has increased rapidly in the last decades and PTMCs account for most new diagnoses. Consequently, an increase in thyroid surgery was observed, but many of thyroidectomies were potentially an overtreatment, as suggested by the unchanged mortality. This scenario allowed the authors to propose active surveillance for PTMCs [26]. However, although evidence suggests that the aggressiveness of PTC is related to the maximum diameter, it is not uncommon to find PTMC in clinical practice with metastases already at diagnosis [27]. Therefore, this study aimed to quantify and investigate clinical factors related to HELNM + in PTMC.

In our multicenter cohort of almost 300 patients with PTC, the overall prevalence of LNM in PTMC was 27.2%. This finding was homogeneous in all three centers involved in the study and is consistent with the figure of another recent study in a large population [9]. This data confirms that although PTC has a generally excellent prognosis, its lymphatic widespread cannot be overlooked also when we look at PTMC. Interestingly, in our study, more than 90% of PTMC HELNM + do not present an aggressive histotype, and the multivariate logistic regression individuates only sex and age as an independent risk factors for LNM in PTMC. In particular, the younger and/or male subjects have greater the risk of having LNM in PTMC. Several previous studies have proposed various clinical characteristics related to metastasis PTMC, but it was never found in full agreement in this regard, excluding male sex and young age. These two factors have gradually emerged as associated to invasiveness of MacroPTC and PTMC [16, 20].

These results merit a full discussion. Histotype should not be considered as the mainstay in evaluating the invasiveness of PTMC, because this feature is not reliable in individuation of cases with LNM and hence it cannot guide the surgery extension. Therefore, strong input emerging from this study concerns the need for efforts in the development of proteomic assays in thyroid cytological samples, rather than genomics, to detect mutated proteins that could promote metastasis. Proteins that characterize mitochondrial inner membrane, mitochondrial transport, cell respiration, and ribosomal activity have already been reported to be more expressed in tissues of PTMC with LNM than those without LNM [28]. Hence, it was hypothesized that mitochondrial dysfunction may promote tumor progression by activating oxidative phosphorylation and PI3K/AKT signaling pathways. Furthermore, four proteins (i.e., SLC25A15, DIRAS2, PLA2R1, and MTARC1) associated with a TC poor prognosis have been recognized to be downregulated in PTMC with LNM; thus, they have been proposed as candidate biomarkers for predicting PTMC metastasis [28].

Given the data of the multivariate regression, in addition to genetic factors, it would be interesting to investigate the hormonal mechanisms that favor cell migration and invasion. To date, studies in this regard are still scant; however, it was already demonstrated that the expression of the estrogen receptors-α and -β, progesterone receptor, and the androgen receptor was higher in PTMCs than in non-tumorous thyroid tissue [29] and that both testosterone and oestradiol have a specific effect on the proliferation of human thyroid papillary carcinoma cell lines independent of TSH action [30, 31]. The young age is related to higher levels of sex hormones. Another potential link of this risk factor with the invasiveness of the TC could be the length of telomeres and the telomerase activity. Thus, mutations of the gene TERT (i.e., telomerase reverse transcriptase) have been considered impactful on TC cell biology and dedifferentiation [32, 33].

In this panorama, active surveillance for PTMCs does not appear to be a truly safe choice for the health of patients and for the healthcare’s economy. In fact, very few PTMC patients, outside the clinical trials, feel confident in choosing an active surveillance approach. Most of them motivate the preference for the early surgical approach with psychological tumour burden [34]. Furthermore, data have already been published demonstrating better long-term cost effectiveness for early PTMC surgery rather than long active surveillance [35].

Finally, a crucial issue has to be discussed. Since according to the guidelines of the last version of the American Thyroid Association [15] and the European Thyroid Association [6] guidelines, thyroid nodules smaller than 10 mm are suitable for FNAC and eventual surgery only if they show significant growth, suspicious lymph nodes, or extrathyroidal extension; it is evident that NEUS plays a crucial role in the correct risk assessment of these lesions. Considering only a dimensional criterion, there is the risk of overlooking a significant percentage of PTMCs that would be incorrectly classified as very-low-risk lesions. Therefore, an evaluation performed by experienced endocrinologists/radiologists able to detect suspicious lymph nodes both at laterocervical and central neck compartment, before surgery, is mandatory for a complete assessment of these patients [36].

The main limit of the study is the retrospective design, which is responsible for the lack of more detailed information about LNM, such as the maximum diameter, the count of dissected lymph nodes, and the LNM involved. Furthermore, genetic evaluations were not performed. However, the population enrolled by three different Italian high-volume institutes allows for a full evaluation that overcomes the regional differences. In addition, all studies in the literature on this topic consider a very broad period (i.e., 10 to 40 years) with a series of biases concerned with technological acquisitions, change of operators, availability of resources for screening, and evolution in approach to disease. Our study was instead designed to perform an actual analysis of the topic by looking at current years.

## Conclusion

In a large national cohort of patients with PTCs, PTMCs with LNM account for almost 30% of cases. Therefore, lymphatic invasion cannot be overlooked. Furthermore, the histological subtype is not reliable in evaluating the invasiveness of PTMC. Finally, this study highlights young age and male sex as the only risk factors for LNM in PTMC. Therefore, in all patients with a small suspicious thyroid nodule, a careful NEUS and clinical evaluation by an expert thyroidologist are mandatory to decide whether surgery and its relative extension is necessary. Further studies are urgently needed on thyroid cell proteomics. International guidelines on PTMC management should consider these issues.

## Data Availability

The datasets used and/or analyzed during the current study are available from the corresponding author upon reasonable request.
